# Bile acid quantification of 20 plasma metabolites identifies lithocholic acid as a putative biomarker in Alzheimer’s disease

**DOI:** 10.1007/s11306-017-1297-5

**Published:** 2017-11-17

**Authors:** Josef Marksteiner, Imrich Blasko, Georg Kemmler, Therese Koal, Christian Humpel

**Affiliations:** 1 Department of Psychiatry and Psychotherapy A, General Hospital, Hall, Austria; 2 Laboratory of Psychiatry and Experimental Alzheimer’s Research, Medical University of Innsbruck, Innsbruck, Austria; 3 Biocrates Life Sciences AG, Innsbruck, Austria; 4 Department of Psychiatry, Psychotherapy and Psychosomatics, Anichstr. 35, 6020 Innsbruck, Austria

**Keywords:** Alzheimer, Diagnosis, Plasma, Bile acid, Lithocholic acid, Biomarkers

## Abstract

**Introduction:**

There is still a clear need for a widely available, inexpensive and reliable method to diagnose Alzheimer’s disease (AD) and monitor disease progression. Liquid chromatography–mass spectrometry (LC-MS) is a powerful analytic technique with a very high sensitivity and specificity.

**Objectives:**

The aim of the present study is to measure concentrations of 20 bile acids using the novel Kit from Biocrates Life Sciences based on LC-MS technique.

**Methods:**

Twenty bile acid metabolites were quantitatively measured in plasma of 30 cognitively healthy subjects, 20 patients with mild cognitive impairment (MCI) and 30 patients suffering from AD.

**Results:**

Levels of lithocholic acid were significantly enhanced in plasma of AD patients (50 ± 6 nM, p = 0.004) compared to healthy controls (32 ± 3 nM). Lithocholic acid plasma levels of MCI patients (41 ± 4 nM) were not significantly different from healthy subjects or AD patients. Levels of glycochenodeoxycholic acid, glycodeoxycholic acid and glycolithocholic acid were significantly higher in AD patients compared to MCI patients (p < 0.05). All other cholic acid metabolites were not significantly different between healthy subjects, MCI patients and AD patients. ROC analysis shows an overall accuracy of about 66%. Discriminant analysis was used to classify patients and we found that 15/23 were correctly diagnosed. We further showed that LCA levels increased by about 3.2 fold when healthy subjects converted to AD patients within a 8–9 year follow up period. Pathway analysis linked these changes to a putative toxic cholesterol pathway.

**Conclusion:**

In conclusion, 4 bile acids may be useful to diagnose AD in plasma samples despite limitations in diagnostic accuracy.

**Electronic supplementary material:**

The online version of this article (10.1007/s11306-017-1297-5) contains supplementary material, which is available to authorized users.

## Introduction

Alzheimer’s disease (AD) is a progressive neurodegenerative disease that gradually leads to severe cognitive deterioration and premature death. The main neuropathological hallmarks of AD concurrently include accumulation of amyloid-β (Aβ) plaques, neurofibrillary tangles (NFT), inflammation and glial responses, synaptic and neuronal loss and vascular alterations. To date, the diagnosis of AD is based on a time-consuming combination of psychological testing, imaging and the analysis of three well-established biomarkers [amyloid-β_42_ (Aβ_42_), total tau and phospho-tau-181] in cerebrospinal fluid (Blennow 2005; Humpel 2011a). The increasing number of early dementia patients forces the identification of economic and reliable biomarkers in blood, urine, or saliva since cerebrospinal fluid collection is an invasive procedure and an expensive analysis (Humpel 2011a; Blennow et al. 2010; Song et al. 2009; Shad et al. 2013; Hu et al. 2012).

Targeted metabolomic analysis in different laboratories including ours allowed to identify several lipid metabolites in plasma being suitable to differentiate AD patients from healthy controls (Mapstone et al. 2014; Olazaran et al. 2015; Klavins et al. 2015). These preliminary data suggested that also bile acids may be altered in AD (Olazaran et al. 2015). Bile acids are major components of bile formed from cholesterol through various enzymatic reactions in hepatocytes. Primary bile acids synthesized in the liver (i.e., cholic acid and chenodeoxycholic acid) in humans are mainly conjugated with taurine or glycine amino acids through the terminal side-chain carboxylic group present in the bile acid structure (Hofmann and Hagey 2008). Bile acids are transported back to the liver via portal blood for re-secretion, a process called enterohepatic bile acid circulation (Li and Chang 2014). Bile acids have multiple roles in humans. Bile acids have hormone-like functions and interact with membrane bound G-proteins coupled receptors of various tissues (Chiang 2002; Lefebvre et al. 2009; Thomas et al. 2008). Bile acids are involved in regulating drug efficiency and toxicity via cytochrome P450 metabolism, and are increased after liver injury (Neale et al. 1971). Bile acids show effects on the gastrointestinal system, and gut microbiota are involved in the transformation of bile acids through deconjugation, dehydroxylation and reconjugation. In fact bile acids can also induce reactive oxygen species and DNA damage (Bernstein et al. 2005). And finally, bile acids are of particular interest because they are the end product of the cholesterol metabolism, which directly link bile acids to AD as cholesterol has been implicated to play a role in progression of AD (Puglielli et al. 2003). In addition to the role in cholesterol elimination, bile acids exert effects on glucose and lipid metabolism via activation of bile acid receptors nuclear-farnesoid-X receptor (FXR) and G-protein-coupled plasma-membrane bound receptors (TGR5), directly linking bile acids with progression of AD with diabetes type II (Schilling 2016) or with cerebrovascular dysfunction (Humpel 2011b).

The aim of the present study is to apply the novel Bile Acid kit from Biocrates to analyze 20 bile acid metabolites. Our data will reveal that plasma levels of the bile acid lithocholic acid (LCA) are significantly different between healthy controls, MCI patients and patients suffering from AD. In a follow up experiment, we demonstrated that LCA levels significantly increased when healthy subjects converted to AD.

## Materials and methods

### Patients

Cognitively healthy subjects and patients suffering from MCI and AD were recruited at Hall/Tirol State Hospital, Austria. For the test set, 80 persons were included in this study, all of whom were older than 70 years (Table 1). For the classification blinded study 23 persons were included. For a conversion study blood was taken from the same of 80 initial patients (n = 8) after 8–9 years and verified that they converted from controls to AD. The procedure for diagnosis has been described by us in detail elsewhere (Hochstrasser et al. 2011, 2012). A panel including a neurologist, psychiatrist and neuropsychologist examined all clinical and diagnostic features. Following a discussion, participants were assigned a diagnosis of MCI or AD according to published criteria (Petersen et al. 2001; McKhann et al. 1984). None of the MCI patients had a score higher than 0.5 in the global score of the Clinical Dementia Rating (Morris 1993). Cognitive functions were assessed with the German version of the CERAD plus neuropsychological battery (Welsh et al. 1994). This test battery provides a reliable profile of cognitive impairments based on demographically adjusted z scores. In addition to eight cognitive domains of CERAD battery, depressive symptoms were assessed using the 30-items version of Geriatric Depression Scale (GDS). All study participants were also assessed by magnetic resonance imaging using a 1.5 T Siemens Symphony MRI scanner with a T1-weighted FLASH 3D sequence and a repetition time (TR) of 9.7 ms, an echotime (TE) of 4 ms, a matrix size of 256 × 256 and a field of view of 230 mm, yielding sagittal slices with a thickness of 1.5 mm and an in-plane resolution of 0.98 × 0.98 mm. Imaging findings were evaluated by a trained radiologist who was blinded to diagnosis and cognitive performance of the patients. Medial temporal atrophy (MTA) was assessed using a standardized scale (Scheltens et al. 1992). Subjects were excluded from the study when suffering from acute viral hepatitis or chronic hepatitis, cirrhosis of the liver, liver damage from alcohol abuse or alcoholic fatty liver; elevations of liver enzymes alanine transaminase (ALT) and aspartate transaminase (AST), other decompensated metabolic diseases and history of drug or alcohol addiction. Participants underwent continuous statin or ezetimibe treatment for at least 3 months before study entry. No patient had a cholesterol level > 240 mg/dL that was not treated with a statin or ezetimibe.The study was approved by the Ethics Committee of the Medical University of Innsbruck and was performed in accordance with the Helsinki Declaration. All subjects gave written informed consent.

**Table 1:** Characteristics of healthy subjects, MCI patients and patients suffering from AD

Diagnostic group	CO	MCI	AD
Sample size	30	20	30
Female/male	22 /8	21/9	11/9
Age (years)	77 ± 1.2	78 ± 1.2	79 ± 2.0
MMSE	29 ± 0.2	28 ± 0.3	20 ± 1.0 ***
GDS	3.3 + 0.5	2.6 ± 0.4	3.1 ± 0.7

Values are given as mean ± SEM

*MMSE* mini-mental state examination (scale 0–30), *GDS* geriatric depression scale (30-items version), *CO* healthy controls, *MCI* mild cognitive impairment, *AD* Alzheimer’s disease

Statistical analysis was performed by One Way ANOVA with a subsequent Fisher LSD posthoc test (***p < 0.001)

### Collection of plasma

Ten mL of EDTA blood was collected, centrifuged (400×*g*, 10 min), and the upper plasma phase was immediately frozen at − 80 °C. Blood processing time was 4.2 ± 0.2 h and did not differ between groups.

### Bile acid analysis and quantification

Analysis was performed using the novel Bile Acids Kit (Biocrates Life Sciences) as described (Pham et al. 2017). The basic parameters are described as follows. The Kit is specified as to measure 16 specific human bile acids and 19 specific rodent bile acids. The total number of bile acids in its panel is 20 as many human and rodent bile acids overlap. In this study the entire panel of the Bile acid Kit is reported. The measurements were carried out on a Shimadzu Nexera X2 UHPLC system coupled with the SCIEX QTRAP 5500 mass spectrometer equipped with an ESI ion source. To ensure accuracy and precision, the Kit provides seven calibration standards, a mixture of 10 isotope-labeled internal standards, and three levels of quality control samples. The calibration range of individual compounds and their assignment of internal standards are given in Supplementary Table 1.

The sample preparation process is as follows. 10 μL of internal standards mixture was pipetted onto the filter spots suspended in the wells of the 96-well filter plate. This filter plate was fixed on top of a deep-well plate serving as a receiving plate for the extract (a combi-plate structure). Subsequently, 10-μL plasma samples were pipetted on the spots, followed by nitrogen drying. Then 100 μL methanol was added to the wells, and the combi-plate was shaken for 20 min. The combiplate was centrifuged to elute the methanol extract into the lower receiving deep-well plate, which was then detached from the upper filter plate. After adding 60 μL Milli-Q^®^ water to the extracts and shaking briefly, the plate was ready for LC-MS/MS analysis. All target isobaric bile acids were baseline separated under ultrahigh pressure liquid chromatography (UHPLC) conditions. UHPLC systems were used at a flow rate of 0.5 mL/min, enabling a short runtime of 5 min. A proprietary reversed- phased UHPLC column (Biocrates Life Sciences) was used. Chromatographic conditions (e.g., mobile phase compositions, gradients, column temperature) were described in details in the provided user manual. Mass spectrometric detection was accomplished with electrospray ionization in negative ion mode. Two MRM transitions are used for each target compound, whereas the more intensive signals are used for quantitation. The weaker MRM transitions are used to confirm the identity of target bile acids, which, in combination with the chromatographic retention times offer a near absolute certainty for target compound identification. Supplementary Fig. 1 shows typical extracted ion chromatograms of a Bile Acids Kit calibrator (upper panel A) and a representative pooled human plasma sample (lower panel B). For the quantification, a calibration set with 7 concentration levels and a mixture of 10 internal standards were used. The calibration regression was set to quadratic with 1/x^2 weighting. An example of a calibration curve for CDCA on for different days (interday experiment), demonstrating the robustness of the assay and stability of (instrument) calibration, is shown in Supplementary Fig. 1C.

The assay has been rigorously validated in accordance with European Medicines Agency (EMA) guidelines. All performance parameters such as selectivity, dilution and spiking integrity through the intra- and interday (n = 6 and n = 4, respectively) precision and accuracy of calibrators and diluted, unspiked as well as spiked human plasma samples, matrix effect, carry-over and short-term stability have been investigated on multiple (U)HPLC-MS platforms. All acceptance criteria laid out in the EMA guideline have been fulfilled after this extensive validation process. The EMA guideline does not require explicitly the determination of the recovery rate of extraction. The recovery rate does not have to be 100% but needs to be constant to guarantee the reproducibility of measurements. This requirement has been indirectly fulfilled through the determination of the accuracy of the spiked human plasma samples. Matrix effects typically occur in MS-based analyses and generally lead to ion suppression of the analyte signals. The matrix effect was investigated in “post-extraction spiking” experiement. Six individual plasma samples have been used, including one lipaemic sample (triglyceride content > 200 mg/dL and cholesterol > 200 mg/dL) and one hemolytic sample (hemoglobin content > 20 g/dL). The samples were spiked after the extraction with bile acid standard solution at two concentration levels. Internal standards were also spiked into the extracts to compensate the matrix effects. Ideally, the suppression of analyte signals is the same as for internal standard signals, so that the ratio of their peak areas remains unchanged. By using 10 different stable isotope-labeled internal standards, it has been proven that matrix effects for the target bile acids in human samples can be effectively compensated. More importantly a proficiency test carried out in 12 different laboratories has shown excellent accuracy (within 85–115%) and precision (CV < 20% for all target bile acids) of the Kit (Pham et al. 2017).

### Statistical Analysis

Bile acid concentrations were compared between groups (control, MCI, AD) by One Way ANOVA and subsequent Fisher LSD post-hoc tests. Bile acids whose concentration differed significantly between groups were resulted into a receiver operating characteristic (ROC) analysis to derive optimal cut-off levels as well as estimates of sensitivity, specificity and the area under the ROC curve. Moreover, linear discriminant analysis with forward stepwise variable selection and equal a priori probabilities for the three groups was used to obtain a classification rule based on bile acid concentrations. Selection of predictor variables was based on Wilk’s lambda and the corresponding F-statistic, entering all variables with a p value < 0.05 into the model. Predicted group membership was determined using Fisher’s linear discriminant functions. Bias-corrected classification rates were obtained by means of cross validation.

## Results

### Patients

The demographic and clinical characteristics for the test dataset of the healthy subjects, MCI and AD patients are given in Table 1. All individuals in the study population are in the same age group between 77 and 79 years and do not differ between groups (Table 1). All groups included more females (Table 1). The MMSE score was 29.0 for controls, not different in MCI but significantly lower in AD patients (Table 1). The GDS values were around 3 and did not differ between groups (Table 1).

### Plasma bile acids analysis

Levels of lithocholic acid were significantly enhanced in plasma of AD patients (50 ± 6 nM, p = 0.004) compared to healthy controls (32 ± 3 nM; Table 2). Lithocholic acid plasma levels of MCI patients (41 ± 4 nM) were not significantly different from healthy subjects or AD patients. Levels of glycochenodeoxycholic acid, glycodeoxycholic acid and glycolithocholic acid were significantly higher in AD patients compared to MCI patients (p < 0.05; Table 2). All other cholic acid metabolites were not significantly different between healthy subjects, MCI patients and AD patients (Table 2). Muricholic acid was below detection limit in all samples (data not shown). The sum of all bile acids was significantly increased in AD patients (Table 2).

**Table 2:** Levels of different bile acids in plasma of cognitively healthy subjects, MCI patients and patients suffering from Alzheimer´s disease

Analyte	DL	Control (n = 30)	MCI (n = 20)	AD (n = 30)	CO versus MCI	CO versus AD	MCI versus AD
CA, cholic acid	10	215 ± 51	155 ± 43	224 ± 161	ns	ns	ns
CDCA, chenodeoxycholic acid	6	361 ± 84	284 ± 61	1245 ± 1009	ns	ns	ns
DCA, deoxycholic acid	6	455 ± 68	291 ± 50	723 ± 236	ns	ns	ns
GCA, glycocholic acid	10	423 ± 145	207 ± 24	332 ± 51	ns	ns	ns
GCDCA, glycochenodeoxycholic acid	6	1326 ± 223	1156 ± 105	1951 ± 301	ns	p = 0.06	p = 0.035
GDCA, glycodeoxycholic acid	3	640 ± 90	504 ± 105	956 ± 180	ns	p = 0.09	p = 0.034
GLCA, glycolithocholic acid	3	41 ± 8	27 ± 4	66 ± 15	ns	ns	p = 0.026
GUDCA, glycoursodeoxycholic acid	3	160 ± 29	137 ± 17	137 ± 24	ns	ns	ns
LCA, lithocholic acid	3	32 ± 3	41 ± 4	50 ± 6	ns	p = 0.004	ns
TCA, taurocholic acid	6	134 ± 64	40 ± 9	99 ± 25	ns	ns	ns
TCDCA, taurochenodeoxycholic acid	3	211 ± 58	170 ± 37	347 ± 71	ns	ns	ns
TDCA, taurodeoxycholic acid	3	97 ± 23	72 ± 20	152 ± 36	ns	ns	ns
TLCA, taurolithocholic acid	3	8 ± 2	5 ± 1	11 ± 2	ns	ns	ns
TMCA(a + b), tauromuricholic acid (sum)	3	15 ± 3	17 ± 5	24 ± 5	ns	ns	ns
TUDCA, tauroursodeoxycholic acid	3	8 ± 1	8 ± 1	11 ± 2	ns	ns	ns
UDCA, ursodeoxycholic acid	6	88 ± 23	62 ± 17	209 ± 149	ns	ns	ns
Sum of all bile acids		4214 ± 419	3176 ± 324	6537 ± 1163	ns	p = 0.031	ns

Values represent nM in mean ± SEM. Statistical analysis was performed by One Way ANOVA with a subsequent Fisher LSD posthoc test where p < 0.05 was considered as significant

*DL* detection limit, *ns* not significant

### Findings of ROC analyses and discriminant analysis

Findings of the ROC analyses are summarized in Table 3. LCA levels differentiated only moderately between AD patients and controls, with an AUC of 0.689 under the ROC curve. For the cut-off level of 42.5 (the statistically best value) a fairly high specificity (0.833) was achieved in connection with rather modest sensitivity (0.5). GDCA and GLCA concentrations discriminated only moderately well between MCI and AD patients with AUC values of 0.688 and 0.654, respectively, and sensitivity and specificity values between 0.6 and 0.7. With an AUC of 0.639, GLDCA showed an even poorer discrimination between MCI and AD patients.

**Table 3:** ROC analysis: discrimination between healthy controls (CO) and patients with Alzheimer’s disease (AD) or mild cognitive impairment (MCI)

Discrimination between CO and AD						
Parameter	Area under ROC curve		Optimal cutoff	Sensitivity	Specificity	Overall accuracy^a^
	Value	95% CI				
LCA	0.689	0.556–0.822	42.5^b^	0.500	0.833	0.667

Discrimination between MCI and AD						
Parameter	Area under ROC curve		Optimal cutoff	Sensitivity	Specificity	Overall accuracy^a^
	Value	95% CI				
GDCA	0.688	0.536–0.839	555^b^	0.667	0.700	0.687
GLCDA	0.639^c^	0.487–0.791	1300^d^	0.600	0.650	0.630
GLCA	0.654	0.503–0.805	31^b^	0.600	0.700	0.660

^a^Proportion of correctly classified cases

^b^Maximizes the sum of sensitivity and specificity

^c^Not significantly different from 0.5 (i.e., not significantly larger than values that may arise from guessing)

^d^Maximizes the sum of sensitivity and specificity under the constraint that both sensitivity and specificity are ≥ 0.6

Results of the discriminant analysis are displayed in Table 4. Of the four bile acids identified as potential predictors only two were entered into the model, LCA (Wilk’s λ = 0.876; d.f. = 1, 2, 77; F 5.45; p = 0.006) and GDCA (Wilk’s λ = 0.797 d.f. = 2, 2, 77, F = 4.56, p = 0.002); GLCA and GLDCA were not entered as they did not significantly improve prediction of group membership. With a total of 47.5% correctly classified cases (45% after bias-correction) overall prediction was only modest. The proportion of slightly misclassified cases (e.g., MCI classified as control or as AD) reached rather high levels (31.3% after bias-correction) while the percentage of completely misclassified cases (AD classified as control or vice versa) was somewhat lower (23.7%). Using discriminant analysis we aimed to classify blinded samples and correctly classified 15 out of 23 (Table 5).

**Table 4:** Discriminant analysis using log-transformed LCA and GDCA as a model (GCDCA and GLCA were not significant and hence not entered into the model)

Classification results			
Group	Predicted group membership		
	Control	MCI	AD
Control (N = 30)	12 (40.0%)	9 (30.0%)	9 (30.0%)
MCI (N = 20)	3 (15.0%)	11 (55.0%)	6 (30.0%)
AD (N = 30)	10 (33.3%)	5 (16.7%)	15 (50.0%)

	Goodness of prediction (in brackets: bias-correction by means of cross-classification)		
	Correct	One category off^a^	Completely misclassified^b^
Control (N = 30)	40.0% (40.0%)	30.0% (30.0%)	30.0% (30.0%)
MCI (N = 20)	55.0% (50.0%)	45.0% (50.0%)	– (–)
AD (N = 30)	50.0% (46.7%)	16.7% (20.0%)	33.3% (33.3%)
Total (N = 80)	47.5% (45.0%)	28.8% (31.3%)	23.7% (23.7%)

*Classification formula*

Control f0 = 6.576* ln(GDCA) + 8.673* ln(LCA) – 35.996

MCI f1 = 5.636* ln(GDCA) + 10.451* ln(LCA) – 36.547

AD f2 = 6.528* ln(GDCA) + 10.356* ln(LCA) – 41.679

*Rule* Classify as control if f0 > f1 and f0 > f2, classify as MCI if f1 > f0 and f1 > f2, classify as AD if

f2 > f0 and f2 > f1

^a^Actual group and predicted group in adjacent categories, e.g., actual group = healthy, predicted group = MCI

^b^Actual group and predicted group are completely different, e.g., actual group = healthy, predicted group = AD

**Table 5:** Classification of blinded samples using discriminant analysis

Nr	Age	Gender	MMSE	GDS	Clinical diagnosis	LCA	GDCA	f0	f1	f2	Classification	Correct
1	84	M	29	4	Healthy	32	206	29.10	29.70	28.99	f1	No
2	76	F	28	16	MCI	43	430	36.50	36.94	36.86	f1	Yes
3	85	M	21	2	Alzheimer	110	5211	61.05	60.81	62.87	f2	Yes
4	76	M	30	6	Healthy	32	482	34.69	34.49	34.54	f0	Yes
5	77	M	29	1	MCI	30	0	15.87	18.17	15.75	f1	Yes
6	87	M	17	2	Alzheimer	170	3220	61.66	62.65	64.23	f2	Yes
7	81	M	30	6	Healthy	44	380	35.89	36.48	36.29	f1	No
8	71	F	27	2	MCI	33	460	34.65	34.55	34.56	f0	No
9	89	F	19	14	Alzheimer	120	3200	58.60	58.97	60.59	f2	Yes
10	79	F	29	3	Healthy	33	271	31.17	31.57	31.10	f1	No
11	71	M	27	2	MCI	32	70	22.00	23.62	21.95	f1	Yes
12	91	F	11	2	Alzheimer	71	929	45.92	46.52	47.08	f2	Yes
13	83	M	29	3	MCI	32	515	35.12	34.87	34.97	f0	No
14	88	F	23	4	Alzheimer	109	624	47.02	48,76	48.92	f2	Yes
15	80	F	16	3	Alzheimer	83	595	44.34	45.64	45.79	f2	Yes
16	82	F	29	1	Healthy	30	259	30.04	30.32	29.82	f1	No
17	79	F	28	7	Healthy	32	682	36.97	36.45	36.81	f0	Yes
18	82	F	29	18	Healthy	31	653	36.41	35.87	36.20	f0	Yes
19	75	F	25	4	Alzheimer	92	557	44.80	46.34	46.42	f2	Yes
20	90	F	16	3	Alzheimer	75	161	34.86	37.21	36.20	f1	No
21	85	M	21	3	Alzheimer	56	551	40.42	41.09	41.21	f2	Yes
22	80	F	23	20	Alzheimer	42	729	39.77	39.67	40.06	f2	Yes
23	77	M	13	0	Alzheimer	33	312	32.10	32.36	32.02	f1	No

*GCDCA* glycochenodeoxycholic acid, *GDCA* glycodeoxycholic acid, *GLCA* glycolithocholic acid, *LCA* lithocholic acid, *M* male, *F* female, age in years, *MMSE* minimental state examination, *GDS* geriatric depression scale

Verfication is based on discriminant analysis as given in Table 4 (f0 = control, f1 = MCI, f2 = AD) using LCA and GDCA. Note that 15/23 samples were correctly diagnosed

### Conversion study

**Fig. 1:**
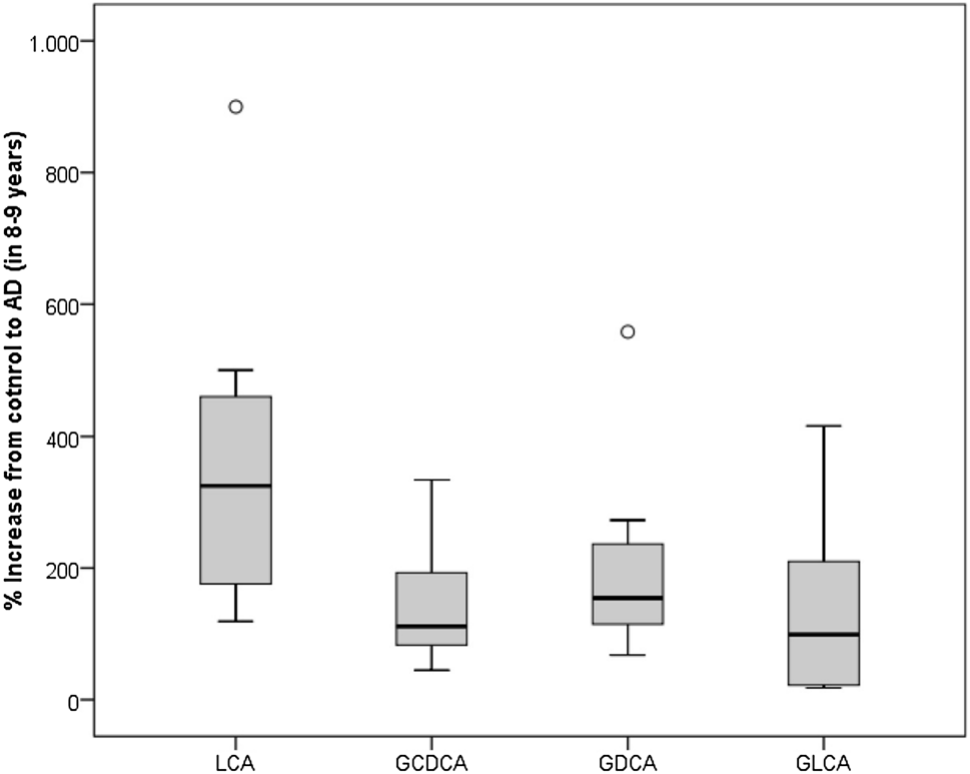
Box-and Whisker Plot showing percent change of LCA, GCCA, GDCA and GLCA plasma levels in 8 converters to AD. Eight healthy control patients were included. Blood samples were recollected after 8–9 years (age 77 ± 3 years, 3 male)

In a follow up experiment, we demonstrated changes of LCA levels in subjects converting from a cognitively healthy status to AD. We included 8 healthy controls. Blood had been collected and had been stored at − 80 °C. After 8–9 years blood was recollected from the same patients who converted to AD. Our data showed that LCA levels increased to 327 ± 100% of the baseline value (t = 0), while all 3 bile acid metabolites (GCDCA, GDCA, GLCA) increased 1.3–2.0-fold in average (Fig. 1).

## Discussion

**Fig. 2:**
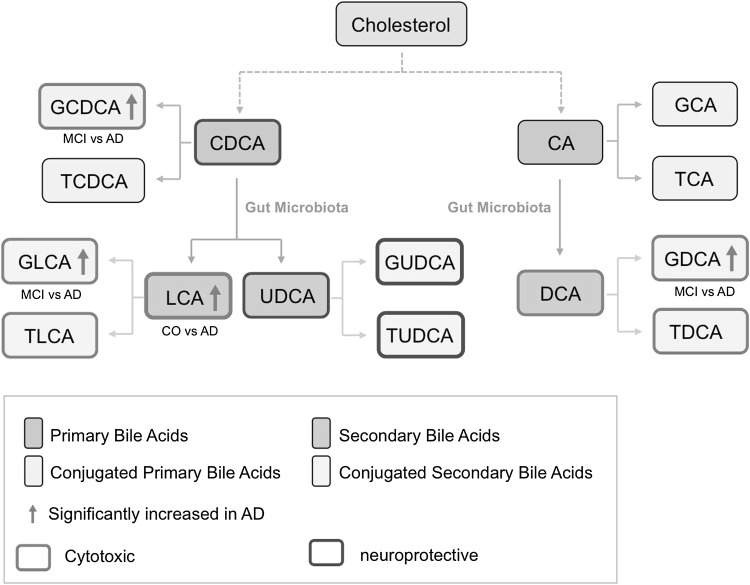
Pathway analysis showing the putative role of the bile acid metabolites in the cholesterol-pathway. Abbreviations see Table 2

In the present study we show that the plasma bile acid lithocholic acid (LCA), as secondary bile acid, differentiates healthy controls from AD patients with a p-value of 0.004. In addition three glycine-conjugated bile acids i.e. GCDCA (conjugated primary bile acid) and GLCA and GDCA (two conjugated primary bile acids) are significantly differentiated AD patients with p values < 0.05. Figure 2 illustrates the appropriate bile acid pathway marked with these up-regulated bile acids.

### Bile acids and AD

Due to their broad physiological function of bile acids in the human body, and their implication in cholesterol, glucose and lipid metabolism there is a clear need to investigate bile acid metabolites in AD. The major bile acid is cholic acid, which can be conjugated or unconjugated forming different isoforms: deoxy-glyco-cheno-lith-hyo-tauro or urso. The nomeclature has been proposed by Hofmann et al. (1992). The novel Bile Acid kit developed by Biocrates allows to measure 20 bile acid metabolites with very high sensitivity (up to 3 nM). Our data not only verify previous preliminary reports on a few bile acid metabolites (Mapstone et al. 2014; Olazaran et al. 2015) but shows for the first time a detailed quantification of up to 20 bile acid metablites in human plasma. Out of these 20 analytes, four metabolites were not detectable, but our data clearly show that bile acids, and especially LCA, are altered in plasma of AD patients. The 3 metabolites GCDCA, GDCA and GLCA were slightly altered, demonstrating the effects on glycine-conjugated bile acids in AD.

### Diagnosis of AD and MCI from plasma by measuring bile acids

Our data show that LCA is highly useful to separate AD patients from healthy controls. Lithocholic acid *(3α-Hydroxy-5β-cholan-24-acid)* is synthesized in the colon from chenodeoxycholic acid and is only reabsorbed in little amounts. LCA has been implicated in carcinogenesis and preliminary data suggests that LCA kills neuroblastoma cells and other malignant cells possibly protecting against colon cancer. LCA (and LCA acetate and LCA propionate) can also activate the vitamin D receptor. In fact, LCA is a very hydrophobic bile acid and is mostly bound to albumin and lipoproteins and may not easily interact with host cells. In addition, the 3 glycine-conjugated bile acids metabolites GCDCA, GDCA and GLCA differentiated AD from MCI patients. Although we cannot give a detailed mechanistic insight into the pathological processes of bile acid metabolism in AD patients, alterations in bile acids may be correlated to the diet of AD patients, reduced physical activity and/or gut microbial composition. Indeed such a gut microbiome may correlate with dehydroxylating gut bacteria, colonic transit time and possibly pH of the colon. A detailed characterization of the gut microbiome of these patients may yield some useful insights, but was out of focus in this study.

We used discriminant analysis for classification of patients. In our verification blinded study with 23 patients of unknown diagnosis we indeed correctly diagnosed 15 subjects (65%). This goes is line with our ROC analysis. Looking at Table 5 it becomes evident that 4 healthy controls, 2 MCI and 2 AD were incorrectly classified. In fact, all healthy controls were classified as MCI cases. This indeed may also point to the problem of incorrect clinical diagnosis. We also are aware that it is not always possible to clearly find good age-matched (70 year old) controls, as we were not able to follow up any comborbidity and additional lifestyle factors, which could influence the clinical diagnosis.

Our conversion study clearly verifies the increase in LCA when controls converted to AD. The plasma LCA increased 3.2-fold within 8–9 years during conversion to AD. There was only 1 patient where we could not measure any LCA in the plasma. The other 3 bile acid metabolites (GCDCA, GDCA and GLCA) only moderately increased during the conversion to AD within the 8–9 years. This again verifies our findings that bile acids are increased in AD. However, when examining bile acid levels within 8–9 years in a longitudinal study it will be of importance to consider what factors changed in their lifestyle. So far, we can exclude that those patients developed another disease, however, we did not evaluate detailed lifestyle parameters. This should be done in a follow-up study.

### Might there be a role of bile acids in AD?

Bile acids are found endogenously in the CSF and brain (Mano et al. 2004; Ogundare et al. 2010). Bile acids are pleiotropic signaling molecules in the body, and the presence of receptors and synthesis enzymes in the CNS indicates that they could be an endogenous signaling system present in the brain. However, clinical studies investigating the dysregulation of the bile acid signaling system in the CNS during stages of Alzheimer´s disease are lacking. There is clear evidence that bile acids play an important role in cardiovascular function: they can modify the vascular tone and can interact with different receptors and transcription factors. It is well known that some cholic acids modulate endothelial cells, cerebral arteries, or aortic smooth muscle cells (Khurana et al. 2011). There is clear evidence that vessels are damaged in AD and that beta-amyloid is deposited in brain vessels (known as cerebral amyloid angiopathy). Although the reasons for development of AD are not know, there is strong evidence that a long lasting chronic vascular impairment (starting decades before onset of AD) plays a role in development of AD (Humpel 2011b). Thus, it is also very likely that such vascular alterations also affect bile acids. A damaged blood–brain barrier causes influx of toxic blood compounds and also entry of blood cells into the brain, but possibly may also result in influx or efflux of bile acids into or from the brain, respectively. Recent evidence also links bile acids with the tumor suppressor gene p53 indicating a role in anti-cancer and apoptosis (Vogel et al. 2012). Indeed, we have shown in our lab, that the tumor suppressor p21 is markedly reduced in monocytes of AD patients (Hochstrasser et al. 2011). Further studies to link bile acids with a damaged blood–brain barrier in AD are necessary.

### Bile acids and the cholesterol-connection

There are several papers published suggesting a link between cholesterol and Alzheimer’s disease (see reviews Anstey et al. 2008; Ribeiro et al. 2015; Reiss and Voloshyna 2012). Also many experiments in rodents have been performed investigating if cholesterol may indeed play a role in development of this disease, including our own research group (Ehrlich and Humpel 2012). This piece of data again supports ideas that indeed the cholesterol pathway may be affected or may play a role in AD. We performed a pathway analysis and our data clearly point to a link between bile acids and cholesterol processing (**see** Fig. 2). Cholesterol is metabolized via different processes into 2 major primary bile acids (CDCA and CA). Both can be conjugated, leading to a putative cytotoxic GCDCA (which is increased in AD in our present study). Modulation of both primary bile acids (CDCA and CA) by the gut microbiota leads to secondary bile acids (LCA, UDCA and DCA), where again the putative cytotoxic LCA is increased in AD in our present study. Conjugation of those secondary bile acids can generate several conjugated forms, including the putative cytotoxic GLCA and GDCA forms, which were both increased in AD in our study. Thus, our data clearly shows that putative cytotoxic bile acids were altered but not putative neuroprotective bile acids (**see** Fig. 2), pointing to a cholesterol-mediated toxicity.

### Limitation of the study

This study can be considered as a pilot study and has definitely clear limitations: A main limitation of the present study is the small size of the samples. This study should therefore be followed up by large-scale multicenter studies that should also include other types of dementia, especially vascular dementia or frontotemporal lobe dementia. In addition, a detailed correlation of ApoE genotype, plasma levels of glucose and insulin, medication, liver enzymes and/or post-prandial intervals may help to follow up the changes at a more mechanistic level, which was, however, not in focus in this study. Another limitation is that we have not yet proven the stability of the bile acids in blood. As we have processed most samples within 4 h, we probably can exclude degradation, because we recently showed that lipid metabolites are at least stable for 24 h (Klavin et al. 2015). This is also true for samples which have been stored for 8 years at − 80 °C and where we cannot exclude linear time-dependent degradation. It has been recognized that nutritional status can influence blood levels of several metabolites, and should be considered when evaluating metabolomic data. Therefore, some authors recommend fasting before blood collection. We have not yet tested the effects of nutrition in this study. More detailed investigation will be necessary in further studies. The ethnicity of patients may have an effect on metabolism; the present study used samples from a group of persons of the same ethnicity. And finally, we observed during the analysis, that the analytical limit of detection of LCA may vary between different sample batches at different days on mass spectrometry analysis due to instrumental variation in performance over the time. Thus, much care must be taken to ensure that the assays are very sensitive and robust.

## Conclusion

In conclusion our data show that the secondary plasma bile acid lithocholic acid differentiates healthy controls from AD patients with a p value of 0.004. Three glycine-conjugated bile acids significantly differentiated AD with p values < 0.05. Thus, these 4 bile acids may be useful to routinely diagnose AD in plasma samples. Our discrimination and ROC analysis revealed, however, a limited specificity, sensitivity and accuracy compared to other published plasma-markers. Thus, these 4 bile acid markers (and more specifically only LCA and GDCA) may be helpful to further diagnose AD in plasma along with a broader pattern of biomarkers.

## Electronic supplementary material

Below is the link to the electronic supplementary material.

Supplementary material 1 (DOCX 22 KB)

Supplementary material 2 (JPG 312 KB)

## Abbreviations

AD: Alzheimers disease
MCI: Mild cognitive impairment
GCDCA: Glycochenodeoxycholic acid
GDCA: Glycodeoxycholic acid
GLCA: Glycolithocholic acid
LCA: Lithocholic acid
MMSE: Minimental state examination
GDS: Geriatric depression scale
