# Supplementary material for: Bile acid quantification of 20 plasma metabolites identifies lithocholic acid as a putative biomarker in Alzheimer’s disease
Source: Metabolomics. 2017 Nov 17;14(1):1. doi: 10.1007/s11306-017-1297-5 (PMC5725507; doi:10.1007/s11306-017-1297-5)
Supplement: Supplementary file 1 — Supplementary material 1 (DOCX 22 KB) [file 11306_2017_1297_MOESM1_ESM.docx]

**Supplemental Table 1**: Compound panel of the Bile Acids Kit. The calibration range covers the normal as well as the abnormal concentration levels to be expected in real samples. LLOQ = Lower Limit of Quantitation, ULOQ = Upper Limit of Quantitation.

| **No** | **Analyte** | **Name** | **Internal Standard (IS)** | **Validity in human** | **Validity in mouse** | **Calibration range**  **(LLOQ – ULOQ)**  **(µmol/L)** |
| --- | --- | --- | --- | --- | --- | --- |
| 1 | CA | Cholic acid | d5-CA | **✔** | **✔** | 0.03 – 75 |
| 2 | CDCA | Chenodeoxycholic acid | d5-CDCA | **✔** | **✔** | 0.02 – 30 |
| 3 | DCA | Deoxycholic acid | d5-CDCA | **✔** | **✔** | 0.02 – 10 |
| 4 | GCA | Glycocholic acid | d5-GCA | **✔** | **✔** | 0.03 – 75 |
| 5 | GCDCA | Glycochenodeoxycholic acid | d4-GLCA | **✔** |  | 0.02 – 20 |
| 6 | GDCA | Glycodeoxycholic acid | d4-GLCA | **✔** | **✔** | 0.01 – 10 |
| 7 | GLCA | Glycolithocholic acid | d4-GLCA | **✔** | **✔** | 0.01 – 5 |
| 8 | GUDCA | Glycoursodeoxycholic acid | d4-GUDCA | **✔** | **✔** | 0.01 – 10 |
| 9 | HDCA | Hyodeoxycholic acid | d4-HDCA(3b) |  | **✔** | 0.01 – 5 |
| 10 | LCA | Lithocholic acid | d4-LCA | **✔** | **✔** | 0.01 – 5 |
| 11 | MCA(a) | Alpha-Muricholic acid | d5-CA |  | **✔** | 0.005 – 5 |
| 12 | MCA(b) | Beta-Muricholic acid | d5-CA |  | **✔** | 0.01 – 10 |
| 13 | MCA(o) | Omega-Murichoclic acid | d5-CA |  | **✔** | 0.005 – 5 |
| 14 | TCA | Taurocholic acid | d5-TCA | **✔** | **✔** | 0.02 – 50 |
| 15 | TCDCA | Taurochenodeoxycholic acid | d5-TCDCA | **✔** | **✔** | 0.01 – 20 |
| 16 | TDCA | Taurodeoxycholic acid | d5-TCDCA | **✔** | **✔** | 0.01 – 10 |
| 17 | TLCA | Taurolithocholic acid | d4-GLCA | **✔** | **✔** | 0.01 – 5 |
| 18 | TMCA(a+b) | Tauromuricholic acid (alpha + beta) | d5-TUDCA | **✔** | **✔** | 0.01 – 10 |
| 19 | TUDCA | Tauroursodeoxycholic acid | d5-TUDCA | **✔** | **✔** | 0.01 – 15 |
| 20 | UDCA | Ursodeoxycholic acid | d4-HDCA(3b) | **✔** | **✔** | 0.02 – 30 |
|  | **Total:** | | **10** | **16** | **19** |  |
