# Supplementary figures and images for: Bile acid quantification of 20 plasma metabolites identifies lithocholic acid as a putative biomarker in Alzheimer’s disease
Source: Metabolomics. 2017 Nov 17;14(1):1. doi: 10.1007/s11306-017-1297-5 (PMC5725507; doi:10.1007/s11306-017-1297-5)

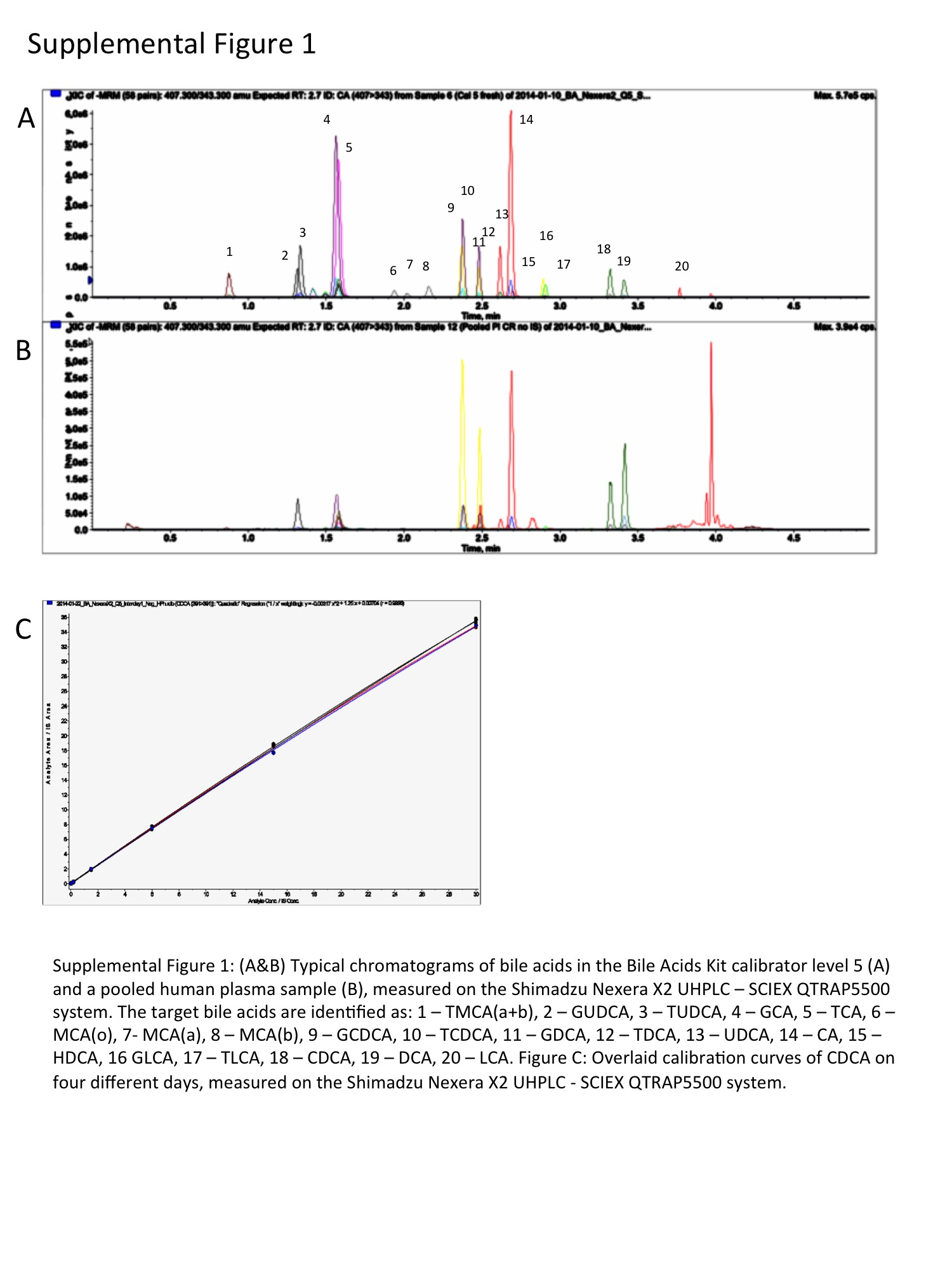

Supplement: Supplementary file 2 — Supplementary material 2 (JPG 312 KB) [file 11306_2017_1297_MOESM2_ESM.jpg]
